# Evaluation of Platelet Distribution Width as an Early Predictor of Acute Kidney Injury in Extensive Burn Patients

**DOI:** 10.1155/2023/6694313

**Published:** 2023-09-07

**Authors:** Ming Jiang, Qingrong Zhang, Chuwei Zhang, Zihan Li, Qiqi Li, Xun Qu, Yi Zhang, Kesu Hu

**Affiliations:** ^1^Department of Burn and Plastic Surgery, Affiliated Hospital of Nantong University, Nantong, Jiangsu, China; ^2^Nantong University Medical School, Nantong, Jiangsu, China; ^3^Third Military Medical University (Army Medical University), Chongqing, China

## Abstract

**Background:**

The extensive burns devastate trauma. The research was designed to analyse the predictive value of early platelet (PLT) indices on the development of acute kidney injury (AKI) after severe burns.

**Methods and Results:**

186 patients with *e*xtensive burns (burn area ≥30%) were eventually involved. Multivariate analyses pointed out that platelet distribution width (PDW) in the first 24 h after admission was an independent risk factor for AKI, severe AKI, and RRT requirement in patients with severe burns, and AKI risk showed an increase of 30.9% per increase of 1% in PDW (OR = 1.309, CI, 1.075–1.594, and *P* = 0.007). It was found that the area under the ROC curve (AUC) of PDW predicting AKI was 0.735 and that the AUC value was 0.81 for AKI after combining PDW and blood urea nitrogen (BUN). Based on the cut-off value PDW = 17.7%, patients were divided into high- (PDW ≥17.7%) and low-risk (PDW <17.7%) groups. In the KM analysis, there was a higher cumulative incidence of AKI if patients were in a high-risk group (in 30 days); and the stages of AKI showed a linear upward trend (chi-square test for linear trend *P*  <  0.001) as there was an increase in the risk level.

**Conclusion:**

The PDW level in the early stage serves as an important risk factor for AKI, severe AKI, and RRT requirement in extensive burns. When PDW >17.7%, burn patients are not only at a higher risk for AKI but may also have higher AKI severity. Due to low cost and wide availability, PDW has the potential to be the tool that can predict AKI in extensive burn patients.

## 1. Introduction

Severe burns are devastating and are linked to a high mortality rate [1]. In the world, it is found that around 11 million people can suffer burns each year, in which a total of 300,000 die [2]. Acute kidney injury (AKI) generally occurs in severely burned patients and has a close linkage with short-term and long-term adverse events. In addition, it can usually cause increased patient mortality and prolonged intensive care [3]. Although early kidney dysfunction may be recoverable, tissue damage is irreversible [4]. There is a high AKI within the general incidence even though recent years have witnessed achievements in intensive care, fluid resuscitation, and renal replacement therapy (RRT) technology [5]. As a result, early prediction and risk stratification of AKI in severe burn patients play a significant role in timely intervention and improved prognosis.

Platelets (PLT) are small, circulating, and anucleate cells derived from megakaryocytes in the bone marrow. Their primary physiological role lies in sensing the damaged vessel endothelium and accumulating at the site of the vessel injury, in which place they can start blood clotting to block the circulatory leak. However, in addition to haemostasis and thrombosis, platelets are included in a lot of diverse biological processes, such as tissue repair, inflammation, and antimicrobial host defense [6].

Platelet indices are investigated as biomarkers in lots of diseases, such as stroke, various cancers, and some infectious diseases [7, 8]. According to Osuka et al. [9], early thrombopenia is an independent risk factor for 60-day mortality within severely burned patients. Recently, Lin et al. [10] pointed out that platelet distribution width (PDW) and PDW-to-platelet ratio (PPR) were good prognostic indicators for mortality in severe burn patients. Also, Wu et al. [11] found that nadir platelet count in the first 2 days of hemorrhagic shock serves as a new biomarker for AKI and 28-day all-cause mortality. However, it is still uncertain whether platelet indices are significant predictors for AKI in burn patients.

As a result, this retrospective study collected the platelet indices on admission and development of AKI in patients with severe burns. The research aimed to validate and evaluate the predictive value of platelet indices of AKI after severe burns. Our work offers a clinical rationale to assess patients' prognosis and enables early intervention timely.

## 2. Methods

### 2.1. Study Population

**Figure 1:**
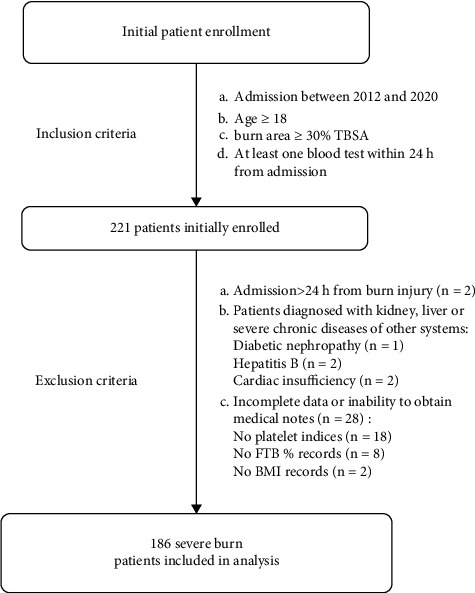
Flowchart of participants presenting the application of inclusion and exclusion criteria. BMI: body mass index; FTBs: full-thickness burns.

The retrospective study was carried out in the Affiliated Hospital of Nantong University. It can be seen in Figure 1 that a total of 221 severe burn patients enrolled at the beginning, of which 35 were excluded, and 186 patients were finally recruited. The research was approved by the Medical Ethics Committee of the Hospital and was following the Helsinki Declaration.

### 2.2. Management and Outcome Definitions

The patient care and treatment abide by the relevant guidelines (such as inhalation injury management, surgery and nutrition, fluid resuscitation, and infection prevention and treatment). On admission, fluid resuscitation was carried out on each enrolled patient in accordance with the Ruijin resuscitation formula which was generally used for China's severe burn patients [12]. The surgery was carried out in patients within 1 month of the injury (escharotomy was performed first, and repeated autologous transplantations were followed). Airway treatment and ventilator assistance were offered to patients with underlying airway blockage or severe hypoxemia. Patients were offered enteral and parenteral nutrition early. Extensive burns were defined as the total body surface area (TBSA) ≥30%. There was an evaluation of AKI severity and incidence according to the Kidney Disease, which were the Improving Global Outcomes (KDIGO) criteria [13]. Severe AKI was defined as AKI stage 2 or 3 [14]. Because the baseline serum creatinine (Scr) levels before injury were often unmeasured in most patients, the first available Scr level was the baseline Scr [5]. The urine output data heterogeneity enables the phenomenon that the assessment of AKI did not involve urine output [15, 16]. The nonrenal sequential organ failure assessment (SOFA) was defined as the SOFA score with no renal component [17]. Life-threatening organ dysfunction can be represented by an increase in the SOFA score of ≥2 points [18].

### 2.3. Data Collection

Each of the laboratory and clinical data was obtained from the hospital's electronic medical record (EMR) system. After admission, patients regularly underwent blood biochemical tests. The results of the blood sample 24 h after admission were chosen to be the research variables. The following variables were gathered, which were age, body mass index (BMI), full-thickness burns (FTBs)%, gender, inhalation injury, tracheostomy, SOFA score, burn mechanism, TBSA%, first 24 h fluid resuscitation volume after injury, MPV (mean platelet volume), PDW, PLT, blood urea nitrogen (BUN), HGB (hemoglobin), ABSI score, the 30-day development of AKI, as well as patients required RRT and in-hospital-survival.

### 2.4. Statistical Analysis

The statistics were explored via the SPSS 23.0, MedCal 20.022, and GraphPad 9.3.1. For exploring the distribution characteristics of each variable, the Shapiro–Wilk test was used. The continuous variables that follow the normal distribution were expressed as the mean ± standard deviation. The *t*-test of the students was applied to show that there were some differences between the two normally distributed variables. In case of no normal distribution, then the continuous variables were expressed as the median (interquartile range (IQR)) and checked through the Mann–Whitney *U* test. By using the chi-square or Fisher's exact test, the categorical data were reported as a percentage and also compared. The chi-square test for trend was used to evaluate the proportionate trends. An exploration of the risk factors that affect AKI was carried out through multivariate logistic regression analysis. The multicollinearity test included the variance inflation factor (VIF) for assessing multicollinearity. For presenting and comparing the predictive values of AKI risk factors, the receiver operating characteristic (ROC) curves were used. We also simultaneously calculated the specificity, sensitivity, and cut-off value. The Youden index was used to decide the cut-off value. The AUC values were compared by the *Z*-test. The Kaplan–Meier (KM) method and log-rank test were used to explore and compare the cumulative incidences of AKI in 30 days. *P* value <0.05 was considered statistically significant.

## 3. Results

### 3.1. Patient Characteristics

The study involved a total of 186 extensively burn individuals. In the cohort, patients had an average age of 48.8, of which the majority was male (67.7%, 126/186). In addition, they also had a median TBSA, FTB, ABSI score, and nonrenal SOFA score of 59%, 12%, 11, and 2 (IQR, 45%–78%, 3%–24.25%, 10–13, and 1–3.25), separately. It is found that flame is the major cause of injury. The research has, respectively, 142, 24, 11, and 5 (76.3%, 12.9%, 5.9%, and 2.7%) patients who suffered from fire burns, chemical and electrical burns, and thermal fluid or steam scalds. In addition, 4 (2.2%) patients had burns due to the existence of some other causes. 146 (78.5%) patients were diagnosed with respiratory burns, of which 98 (52.7%) required tracheostomy. During the first 24 hours, the median fluid resuscitation volume was 366.35 ml/kg (IQR, 259.11 ml/kg–458.33 ml/kg). Furthermore, the median level of early PDW, MPV, PLT, HGB, and BUN were 16.3%, 10.7 fl, 159.5 × 10^9^/L, 162.0 g/L, and 6.0 mmol/L (IQR, 14.3%–17.4%, 9.68 fl–11.9 fl, 119 × 10^9^/L–196 × 10^9^/L, 145.75 g/L–179 g/L, and 4.88 mmol/L–7.6 mmol/L), separately.

For the 186 patients, there was AKI in 41 (22%) during the hospitalization in 30 days following the KDIGO guidelines. There were 14, 17, 10, and 27 patients with stages 1 (7.5%), 2 (9.1%), 3 (5.4%), and severe AKI (14.5%), separately. 8 patients received RRT. 128 (68.8%) patients see their SOFA score ≥2. While hospitalized, 23 (12.4%) patients in total died.

Table 1 shows the comparison of the baseline and demographic differences between AKI and non-AKI groups. In terms of BMI, age, gender, PLT, MPV, and burn mechanism, it is found that no obvious difference was between AKI and non-AKI groups (*P*  >  0.05). In comparison with the non-AKI ones, it is found that AKI patients were seen to have significantly higher TBSA, FTBs, ABSI, nonrenal SOFA scores, the proportion of patients with the SOFA score ≥2, first 24 h fluid amount, inhalation injury proportion, BUN, HGB, PDW, and mortality (*P*  >  0.05).

### 3.2. Logistic Regression Analysis

**Figure 2:**
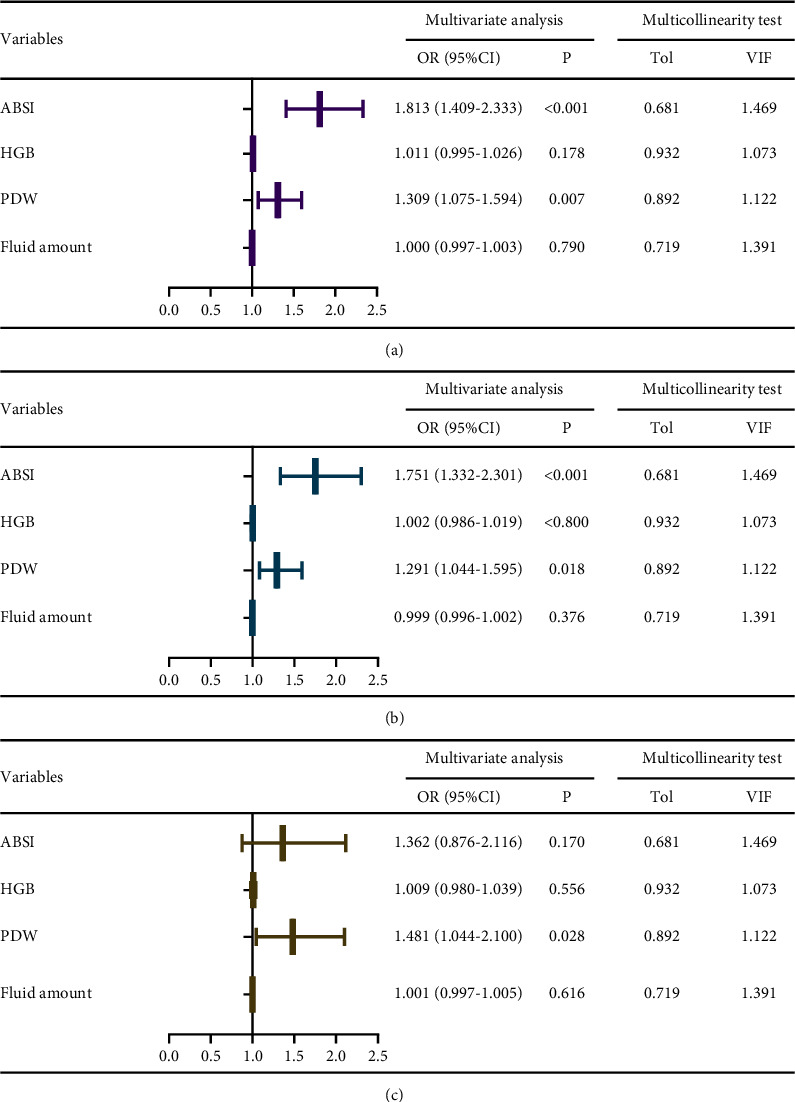
Results of multivariate logistic regression analysis for AKI (a), severe AKI (b), and RRT requirements (c). ABSI: abbreviated burn severity index; HGB, hemoglobin; PDW: platelet distribution width; OR: odds ratio; CI: confidence interval; Tol: tolerance; VIF: variance inflation factor.

Among the platelet indices, only PDW is the significant risk variable for AKI (Table 1). Then, the multivariate logistic regression analysis was applied to evaluate the PDW, HGB, first 24 h fluid amount, and ABSI score for the AKI, severe AKI, and RRT requirements (ABSI primarily combines data on the age, TBSA, FTBs, gender, and inhalation injury of the burn patients and presents the basic characteristics and severity of the burn patients on admission. Therefore, the ABSI score was added to adjust the burning burden in the multivariate analysis. Hemoglobin concentration in blood was taken as a marker of hemoconcentration, which may affect the outcomes. The multicollinearity test showed no obvious linearity between clinical outcomes and factors, all VIF <5 (Figure 2). Figure 2 showed the analysis results. After the multivariate analyses, it was found that PDW represented an independent risk factor for AKI (OR = 1.309, CI, 1.075–1.594, and *P* = 0.007), severe AKI (OR = 1.291, CI, 1.044–1.595, and *P* = 0.018), and RRT requirements (OR = 1.481, CI, 1.044–2.100, and *P* = 0.028). For each increase of 1% in PDW, the risk of AKI showed an increase of 30.9%.

### 3.3. ROC Curve Analysis

**Figure 3:**
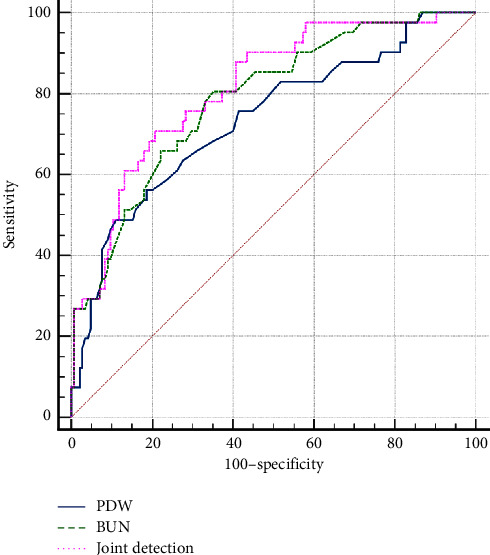
Receiver operator characteristic curves of PDW and BUN and the joint detection factor of PDW and BUN for the prediction of AKI.

The ROC curves were used to evaluate the ability of PDW, BUN (some research see BUN as the related factor for AKI occurrence [19–21]), and the joint detection of BUN and PDW in predicting AKI. When predicting AKI (Figure 3), the AUC of PDW was 0.735, which was very close to the value of BUN (AUC = 0.785). There was no statistically significant difference (*Z* = 0.912 and *P* = 0.361). The cut-off value of PDW was 17.7% (sensitivity: 48.8, specificity: 89.0). There was a higher AUC of 0.813 in the combined test in comparison with PDW and BUN (*Z* = 2.150 and 1.177, *P* = 0.031 and = 0.239, separately).

### 3.4. KM Analysis

**Figure 4:**
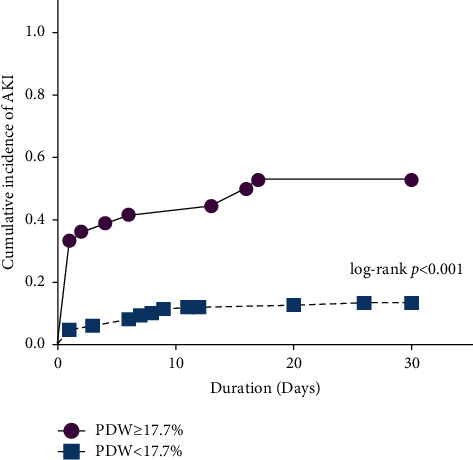
KM curve analysis of AKI development within 30 days based on PDW ≥17.7% and PDW <17.7% in severe burn patients.

The cut-off value of PDW (PDW = 17.7%) was used to classify the patients into high-risk (PDW ≥17.7%) and low-risk (PDW <17.7%) groups. The Kaplan–Meier method was used to identify the 30-day cumulative rate of AKI in different groups (Figure 4). There were significantly higher cumulative incidence curves of PDW ≥17.7% compared to those of PDW <17.7%. Accordingly, there was a higher cumulative incidence of AKI if PDW ≥17.7.

### 3.5. Risk Groups in Different AKI Stages

**Figure 5:**
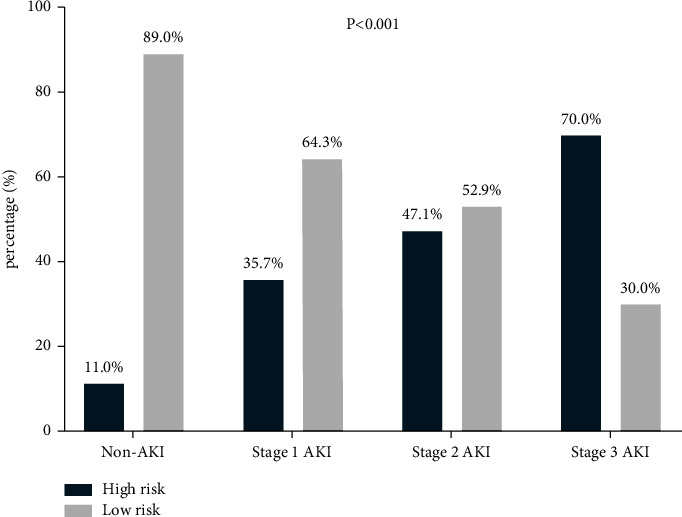
The percentages of high-risk and low-risk groups in different AKI stages. AKI staging presented a linear upward trend as there was an increase of the risk level (the chi-square test for trend *P*  <  0.001).

Figure 5 compared percentages of the high-risk and low-risk groups in different AKI stages. The proportion of the low-risk group (89.0%) in patients without AKI is much higher than that of the high-risk group (11.0%). At the same time, the proportion of the low-risk group (30.0%) in stage 3 AKI patients is significantly lower than that of the high-risk group (70.0%), with all *P*  <  0.05. On the whole, AKI staging presented a linear upward trend as there was an increase in the risk level (the chi-square test for linear trend *P*  <  0.001).

## 4. Discussion

Recently, platelet indices have been proposed as the predictors of thrombotic and inflammatory conditions, which were mainly in patients with thrombotic and inflammatory conditions, and were considered as risk factors for reduced survival in patients with different kinds of malignancies, including non-small cell lung cancer, cervical cancer, pancreatic adenocarcinoma, and colon cancer [7, 8, 22]. Also, their relationship with extensive burns was investigated. According to Cato et al. [23], platelet count and rBaux score together generate moderate discriminatory power for survival at less than 24 h after injury. Huang et al. [24] reported that blunted daily increase in PCs (platelet counts) is linked to an increase in 30-day mortality. Similarly, Lin et al. [10] found that early postburn PDW was an independent risk factor for 120-day mortality in severe burn patients. In these studies, an association between platelet indices and burn mortality was revealed and prognostic information for the mortality risk assessment in severely burned patients was provided. However, burn patients mainly died of multiple organ failure, and there is no report concerning a prognostic association between platelets and AKI in burn patients.

In this study, it is shown that the early PDW level may be a valuable predictor for AKI after severe burns. The major findings are as follows: (1) early PDW after admission was an independent risk factor for AKI, severe AKI, and RRT requirement in extensive burns even after multivariate logistic regression analyses with HGB, first 24 h fluid amount, and ABSI score. The risk showed an increase of 30.9% for AKI occurrence given per increase of 1% in PDW; (2) the AUC value of PDW was 0.735 in predicting AKI. This result was not statistically significant compared with 0.78 of BUN. Their combined application had a higher AUC value (0.81) upon predicting AKI; (3) the KM analysis showed that AKI had higher cumulative incidence if PDW ≥17.7%; and (4) the severity of AKI presented a linear upward trend when the risk level increases (chi-square test for trend *P*  <  0.001). In the abovementioned results, the potential predictive power and the specific predictive values of the early postburn PDW level for AKI were shown. Using the cut-off value (PDW = 17.7%), it is better to be able to distinguish patients at different risks.

AKI in the early stage of extensive burns can be brought by denatured proteins, cardiac insufficiency, hypovolemia, and inflammatory factors produced by tissue destruction and nephrotoxicity caused by drugs [25–27]. Severe sepsis is often seen as a significant reason for the occurrence of advanced AKI [28, 29]. For a long time, hypovolemia and inadequate fluid resuscitation were seen to be the main factors of early AKI. However, the previous study showed that AKI is likely to still occur after enough fluid resuscitation [30]. Besides, the research on critical care patients shows positive fluid intake is likely to negatively affect renal function and mortality [31–33]. In the results, the shock degree brought by the initial burn damage and the following generation of mass inflammatory factors is likely to be a major reason for AKI in burns. Therefore, the indicators which reflect the conditions of tissue perfusion and original burn severity may be adequate predictors of AKI [34]. In this study, it was found that AKI patients had been given significantly more fluid volume in 24 hours than non-AKI patients (Table 1). The higher and abnormal hemoglobin values in AKI patients suggest that there may be a greater degree of blood concentration and fluid loss after injury, which may influence the development of AKI in patients. However, after multiple regression analysis that involves these variables, it was found that PDW was still an independent risk factor for AKI (Figure 2).

Platelets behave as a major role in coagulation and inflammation and are also involved in acquired and innate immune responses. According to Van Linden et al. [35], thrombocytopenia was a significant risk factor for postoperative AKI after the aortic valve implantation. As pointed out by Kertai et al. [36], the platelet nadir after the coronary artery bypass grafting had a significant association with postoperative AKI. However, this situation may be more special in patients with severe burns. The platelets are consumed in the burn wound, and due to this, dermal vasculature and subsequent microthrombi formation are destructed. The microthrombi form in 24−48 h. The permeability of surrounding vessels shows an increase and is likely to cause increased activation of platelets via interacting with the tissue factors on the subendothelium and activated clotting factors [37, 38]. The activation of platelets brings pseudopodia formation and some other morphological changes. In the platelet activation, some morphological alterations can be caused; that is, it seems that activated platelets are larger by becoming spherical and forming pseudopodia. Accordingly, platelets with enhanced pseudopodia number and size will be different in size that brings about alterations in PDW [39]. In another research [40], a high PDW value showed a broad range of PLT volume, which was brought about by destruction, swelling, and immaturity. This means that a higher PDW value enables more obvious PLT damage and immaturity. Therefore, early PDW can not only reflect the severity of burns but also the formation of microthrombi in the microcirculation, thus reflecting the perfusion status of tissues and becoming an AKI predictor.

However, there are some limitations in this study. First of all, in this single-center retrospective study, a preliminary exploration of the relationship between platelet parameters and AKI in extensive burn patients was carried out. However, there is a limited number of AKI (41/186) and severe AKI (27/186), especially for RRT-requiring patients (8/186). The relatively small number of patients may result in statistical instability. Thus, multicenter prospective research with sample size expansion and further validation should be made. Secondly, Scr is mainly applied to decide the AKI stage and presence. We designated the first available Scr as the baseline Scr since most patients had no baseline Scr measured before injury [41]. This is likely to influence results. At last, our research group did not involve children since they are not yet physically developed, and therefore, further research can be carried out within the underage population.

## 5. Conclusion

In the research, early PDW after admission represented an independent risk factor for AKI, severe AKI, and RRT requirement in extensive burns, and the risk of AKI showed an increase by 30.9% per increase of 1% in PDW. Compared to a single variable applied for prediction, there was a higher predictive value for AKI development within PDW combined with BUN. If PDW >17.7%, burn patients are at a higher risk for AKI and are likely to have higher AKI severity. Through low cost and wide availability, it can be seen that PDW has the potential to be the tool that can predict AKI in extensive burn patients.

## Figures and Tables

**Table 1:** Differences between AKI and non-AKI groups.

	AKI	Non-AKI	*P*
Age (years)	48.80 ± 14.70	48.79 ± 12.05	0.134
BMI (kg/m^2^)	24.42 ± 2.36	24.26 ± 3.27	0.903
Gender, *n*, (%)			0.222
Male	31 (75.6)	95 (65.5)	
Female	10 (24.4)	50 (34.5)	
TBSA (%)	86.0 (72.0–91.5)	55 (43–70)	<0.001
FTB (%)	38.0 (17.0–53.0)	7.0 (2–16.75)	<0.001
Respiratory tract burn	41 (100)	105 (72.4)	<0.001
Tracheotomy	31 (75.6)	67 (46.2)	<0.001
ABSI	14 (13–15)	11 (10–12)	<0.001
None-renal SOFA score	4 (3–6)	2 (1–3)	<0.001
SOFA score ≥2, *n*, (%)	37 (90.2)	91 (62.8)	<0.001
First 24 h fluid amount (ml/kg)	432.87 (332.90–531.31)	340 (253.02–437.17)	0.004
Burn mechanism			0.083
Flame	33 (80.5)	109 (75.2)	
Scald	2 (4.9)	22 (15.2)	
Chemical	3 (7.3)	8 (5.5)	
Electrical	3 (7.3)	2 (1.4)	
Other burn factors	0 (0)	4 (2.8)	
PLT (10^9^/L)	139 (94.5–191.5)	164 (127–197.5)	0.060
MPV (fl)	10.7 (9.35–12.05)	10.7 (9.8–11.9)	0.901
PDW (%)	17.5 (16.3–18.95)	16.1 (14.0–16.9)	<0.001
BUN (mmol/L)	7.9 (6.5–11.25)	5.7 (4.3–7.0)	<0.001
HGB (g/L)	174 (158.5–191)	159 (144.5–176)	0.016
Non-survivors	22 (53.7)	1 (0.7)	<0.001

*Note.* Data are presented as the mean ± SD, median (interquartile range), or number (%). BMI, body mass index; AKI, acute kidney injury; TBSA, total burn surface area; FTBs, full-thickness burns; ABSI, abbreviated burn severity index; SOFA, sequential organ failure assessment score; PLT, platelet; MPV, mean platelet volume; PDW, platelet distribution width; BUN, blood urea nitrogen; HGB, hemoglobin.

## Data Availability

All data included in this study are available upon request from the corresponding author.
